# The transient expression of miR-203 and its inhibiting effects on skeletal muscle cell proliferation and differentiation

**DOI:** 10.1038/cddis.2014.289

**Published:** 2014-07-17

**Authors:** W Luo, H Wu, Y Ye, Z Li, S Hao, L Kong, X Zheng, S Lin, Q Nie, X Zhang

**Affiliations:** 1 Guangdong Provincial Key Lab of Agro-Animal Genomics and Molecular Breeding, and Key Lab of Chicken Genetics, Breeding and Reproduction, Ministry of Agriculture, South China Agricultural University, Guangzhou, Guangdong 510642, China; 2 Department of Animal Genetics, Breeding and Reproduction, College of Animal Science, South China Agricultural University, Guangzhou, Guangdong 510642, China; 3 Department of Veterinary Biomedicine, College of Veterinary Medicine, South China Agricultural University, Guangzhou, Guangdong 510642, China; 4 Department of Animal Science, College of Life Science, Foshan University, Foshan, Guangdong 528231, China

## Abstract

Previous studies have shown that miR-203 is a skin-specific microRNA (miRNA) with a profound role in skin cell differentiation. However, emerging microarray and deep sequencing data revealed that miR-203 is also expressed in embryonic skeletal muscle and myoblasts. In this study, we found that miR-203 was transiently upregulated in chicken embryos on days 10 to 16 (E10–E16) and was sharply downregulated and even not expressed after E16 in chicken embryonic skeletal muscle. Histological profiles and weight variations of embryo skeletal muscle revealed that miR-203 expression is correlated with muscle development. *In vitro* experiments showed that miR-203 exhibited downregulated expression during myoblast differentiation into myotubes. miR-203 overexpression inhibited myoblast proliferation and differentiation, whereas its loss-of-function increased myoblast proliferation and differentiation. During myogenesis, miR-203 can target and inhibit the expression of *c-JUN* and *MEF2C*, which were important for cell proliferation and muscle development, respectively. The overexpression of *c-JUN* significantly promoted myoblast proliferation. Conversely, knockdown of *c-JUN* by siRNA suppressed myoblast proliferation. In addition, the knockdown of *MEF2C* by siRNA significantly inhibited myoblast differentiation. Altogether, these data not only suggested that the expression of miR-203 is transitory during chicken skeletal muscle development but also showed a novel role of miR-203 in inhibiting skeletal muscle cell proliferation and differentiation by repressing *c-JUN* and *MEF2C*, respectively.

Skeletal muscle development is a multistage process involving genetic regulation and environmental cue guidance.^1, 2, 3^ During skeletal myogenesis, muscle precursor cells (myoblasts) differentiate from the somite, migrate to the limb buds and begin to express specific myogenic transcription factors, resulting in the differentiation of myoblasts into myotubes.^4, 5^ When myoblasts initiate the differentiation process, the proliferating myoblasts pause the cell cycle, migrate and adhere to each other, and then fuse to form multinuclei myotubes.^6^ In addition, this differentiation process also occurs during postnatal growth and regeneration of adult skeletal muscle, which is dependent on the activation of muscle satellite cells.^7^

MEF2C belongs to the myocyte enhancer factor 2 family (MEF2) comprising four members encoded by different genes—*MEF2A, B, C* and *D*.^8^ In *Drosophila*, mutations in *MEF2* result in a complete loss of muscle differentiation,^9, 10, 11^ and more than 200 target genes and over 650 regions of the genome are bound by MEF2 during myogenesis,^12^ demonstrating its central role in muscle development. During mouse and chick embryogenesis, MEF2C is the first MEF2 family member to be expressed,^13^ but it has no impact on myoblast specification.^10, 11^ Loss-of-function mutations of *MEF2C* in mice result in embryonic lethality because of the cardiac malformation,^14^ and skeletal muscle-specific deletion of *MEF2C* will cause improper sarcomere organisation and weakened M lines.^15^ In addition, as a co-activator, MEF2C can interact with MAML1, Notch3 and the p38 MAPK pathway, thereby promoting skeletal muscle differentiation.^16, 17, 18^ As transcription factors, many muscle-specific genes, as well as muscle-specific microRNAs (miRNAs) such as miR-1, miR-133 and miR-206, have been shown to be regulated by MEF2C.^18, 19^

c-JUN, a transcriptional activator of the AP-1 family, plays positive roles in cell proliferation and cell cycle progression. A mouse bearing a *c-JUN* null mutation will die at mid-gestation because of the retardation of cell proliferation, and the primary fibroblasts from this *c-JUN* null embryo show greatly reduced growth rates in culture.^20^ *c-JUN* gain-and-loss-of-function reveals that the positive role of c-JUN in cell cycle progression is p53 dependent.^21^ *p53* is an important tumour suppressor gene that has an inhibitory role in cell cycle progression through its target gene, the cyclin-dependent kinase inhibitor *p21.*^22^ c-JUN inhibits *p53* transcription by direct binding to a variant AP-1 site in the *p53* promoter, thereby repressing *p53* expression and inducing cell cycle progression.^21^

Recently, several miRNAs, particularly miR-1, miR-206 and miR-133, were found to participate in muscle development.^23, 24^ These myogenic miRNAs (myomiRs) regulate muscle development by fine-tuning gene expression or acting as binary on/off switches.^25^ To better understand this regulation, we previously counted all of the verified miRNAs involved in skeletal muscle differentiation.^26^ Through interactions with many myogenic regulators, these miRNAs have important roles in the regulation of skeletal muscle differentiation. However, none of these miRNAs were found in a chicken embryo study, which is a classic model for development research.

miR-203 is widely known as a tumour suppressor and skin-specific miRNA.^27^ It can directly repress the expression of the ‘stemness-maintaining' transcription factor p63 during epidermis stratification and differentiation, thus restricting proliferative potential, inducing cell cycle exit and finally promoting epidermal differentiation.^28, 29^ In rhabdomyosarcoma cell, re-expression of miR-203 can inhibited cell growth and promoted myogenic differentiation.^30^ However, none of the previous studies have noticed its expression and roles in skeletal muscle development. In C2C12 myoblasts,^23^ quail myoblasts^31^ and chicken skeletal muscle,^32^ the expression of miR-203 can be detected by microarray, quantitative polymerase chain reaction (qPCR) or deep sequencing. In addition, our previous study found that the expression of miR-203 was highly enriched in chicken embryonic skeletal muscle.^33^ Interestingly, miR-203 expression can be detected in embryonic leg muscle, but the expression was not observed in adult leg muscle.^33^ In addition, miR-203 shows lower expression in fast-growing, heavy-mass chicken skeletal muscle.^32, 33^ In the present study, we explored the functional significance of miR-203 in chicken skeletal muscle development and found that miR-203 could repress primary myoblast proliferation and differentiation by directly inhibiting *c-JUN* and *MEF2C* expression, respectively. Our results confirm and illustrate that a skin-specific miRNA can also express and function in skeletal muscle development.

## Results

### miR-203 expression correlates with chicken embryonic skeletal muscle development

**Figure 1:**
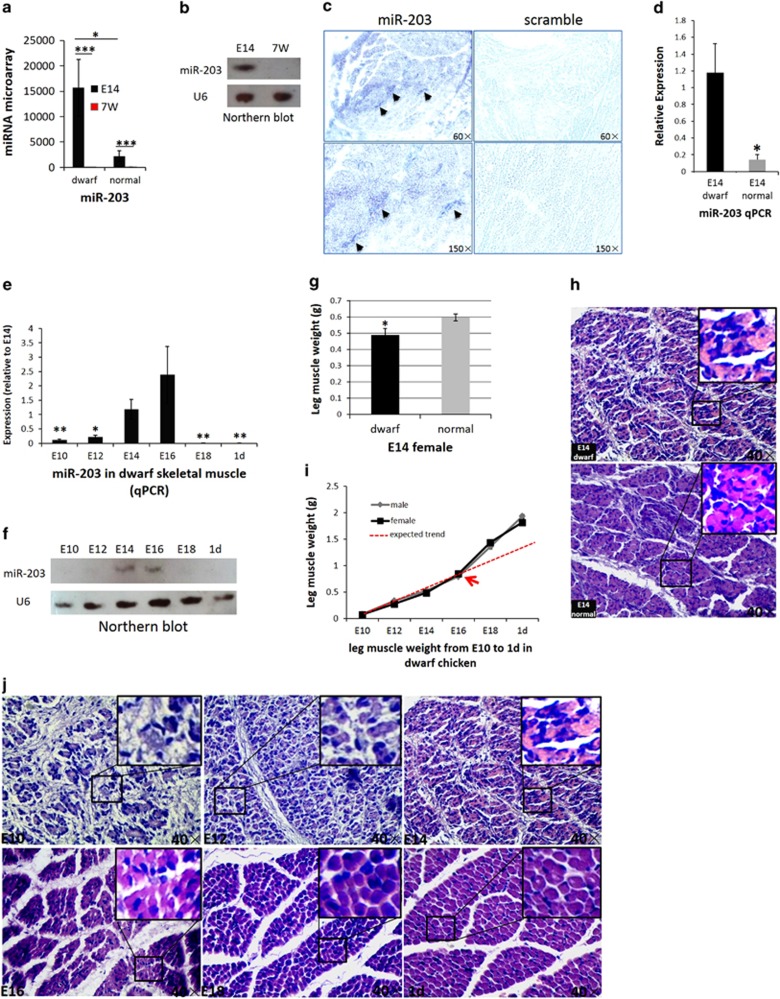
miR-203 expression is correlated with chicken embryonic skeletal muscle development. (**a**) miRNA microarray indicates that miR-203 has abundant detection signals in E14 leg muscles of dwarf and normal White Rock Chickens (black bars). However, the signals were very low in 7-wk-old chickens (red bars). E14 represents an embryo at day 14. 7W represents a chick that was 7 weeks of age. (**b**) Northern blot analysis validated that miR-203 is expressed in E14 leg muscle of dwarf chickens but not in that of the 7-wk-old dwarf chickens. U6 was used as the reference gene. (**c**) *In situ* hybridisation of dwarf chicken E14 leg muscles using anti-miR-203 or scramble confirmed the presence of miR-203 in muscle tissue. Dark blue staining shown by arrows indicates miR-203-positive hybridisation signals. (**d**) The expression of miR-203 is significantly higher in the E14 leg muscle of dwarf chickens than in that of normal chickens. miR-203 relative expression was determined by qPCR. (**e**) The expression of miR-203 in the leg muscle of embryonic dwarf chickens was determined by qPCR. The ages of the chickens were embryo day 10, embryo day 12, embryo day 14, embryo day 16, embryo day 18 and chick at postnatal 1 day of age. The fold change in miR-203 expression, normalised by U6, is expressed relative to E14 miR-203 expression. The results are expressed as the mean±S.E.M. of three replicates. *P<0.05; **P<0.01; ***P<0.001. (**f**) Northern blot analysis validation of miR-203 expression in the leg muscle of embryonic dwarf chickens. (**g**) The E14 leg muscle weight of normal and dwarf female chickens. The E14 leg muscle weight of normal chickens is significantly heavier than that of dwarf chickens. (**h**) H-E staining of histological sections of representative leg muscle from E14 dwarf and normal chickens (magnification, × 40). (**i**) The male and female leg muscle weights of dwarf chickens were collected and measured from embryos at different stages. The red dashed line was placed according to the growth trend from E10–E16, and the red arrows represent an inflection point of muscle growth. Muscle weight is increased faster after E16. (**j**) H-E staining of leg muscle fibre cross section from E10 to 1day in dwarf chickens (magnification, × 40)

Our previous microarray data showed that the expression level of miR-203 is significantly higher in the leg muscle of embryonic day 14 (E14) dwarf chickens than in E14 normal chickens (Figure 1a).^33^ Surprisingly, miR-203 shows abundant detection signals at E14 in both types of chickens but is not expressed at the age of 7 weeks (7 wk; Figure 1a). Considering that miR-203 is specifically expressed in the skin, its high expression level in embryonic leg muscle can be due to the pollution of skin tissue. To eliminate this possibility, we collected other leg muscle samples from E14 and 7 wk dwarf and normal chickens by carefully removing the skin tissue and outer muscle. Subsequently, we used *DSP*, which is specifically expressed in skin tissue, as an RT-PCR marker to further eliminate the pollution of skin RNA (Supplementary File 3). Northern blot analysis showed that miR-203 is indeed expressed in the leg muscle of E14 chickens but is not expressed in the leg muscle of 7-wk-old chickens (Figure 1b). Importantly, miR-203 *in situ* hybridisation in dwarf E14 leg muscle also confirmed its expression in muscle tissue (Figure 1c).

In addition, miR-203 has a significantly higher expression level in dwarf chickens than in normal chickens (Figure 1d), a finding that is consistent with our previous microarray data.^33^ Furthermore, during skeletal muscle development in dwarf chickens, miR-203 expression is upregulated from E10–E16 and sharply downregulated after E16 (Figure 1e). The high expression level of miR-203 at E14 and E16 was also validated using Northern blot (Figure 1f), although the low expression of miR-203 could not be detected at other stages due to the low sensitivity of the assay. In normal chickens, miR-203 is highly expressed at E12 and E14, and miR-203 expression is sharply downregulated after E18 (Supplementary File 1). Altogether, these results confirmed that miR-203 is expressed in chicken embryonic skeletal muscle and indicated that it might have potential roles in skeletal muscle development.

### Histological profiles of muscle fibres and weight variations of skeletal muscle during chicken embryonic development

Gene expression can be linked to organismal phenotypes.^34^ To better understand the potential role of miR-203, we next studied the skeletal muscle phenotypes during chicken embryonic development and observed their correlation with miR-203 expression. Normal chickens have heavier E14 leg muscle weight than dwarf chickens (Figure 1g), and haematoxylin-eosin (H-E) staining showed that the muscle fibres are clear and plump in normal chickens but blurry and flat in dwarf chickens (Figure 1h), suggesting that leg muscle development in dwarf chickens is slower than that of normal chickens. During dwarf chicken leg muscle development, miR-203 exhibits transient upregulated expression from E10–E16 but is sharply downregulated after E16. Therefore, we studied the histological profiles and weight variations of these leg muscle samples. The weights of leg muscles were increased slowly from E10–E16 but were sharply increased after E16 (Figure 1i). H-E staining showed that the structure of the muscle fibres was blurry and irregular during E10–E16, with clear and obvious fibre cross sections in E18 (Figure 1j and Supplementary File 2). Together with the miR-203 expression data, we found that higher or upregulated expression of miR-203 is accompanied by slow leg muscle development, and the low expression of miR-203 is followed by a rapid increase in muscle weight and full formation of muscle fibres. These findings suggested that miR-203 might have an inhibitory role in skeletal muscle development.

### The expression of miR-203 is upregulated in proliferating myoblasts and is sharply downregulated during differentiation *in vitro*

**Figure 2:**
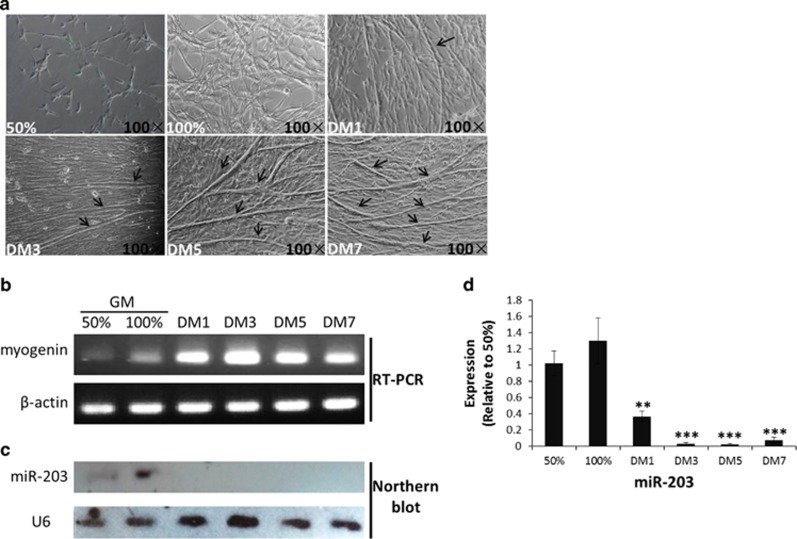
The expression of miR-203 is upregulated in proliferating myoblasts and is sharply downregulated during differentiation *in vitro*. (**a**) Microscopic images of chicken primary myoblasts cultured in GM (50% and 100% confluency) or in differentiation medium (DM) for 1, 3, 5 and 7 days (DM, DM3, DM5 and DM7). Black arrows represent myotubes. (**b**) RT-PCR for detecting myogenin mRNA expression. *β*-actin was used as the reference gene. (**c**) Northern blot analysis for the expression of miR-203 during myoblast differentiation into myotubes. (**d**) miR-203 expression is determined by real-time PCR in chicken primary myoblasts cultured as described in (**a**). The results are expressed as the mean±S.E.M. of three replicates. *P<0.05; **P<0.01; ***P<0.001

To further study the potential roles of miR-203, we separated chicken primary myoblasts (Figure 2a) and observed the expression profiles of miR-203 *in vitro*. miR-203 was expressed in undifferentiated primary myoblasts at both subconfluence (50% growth medium (GM)) and full confluence (100% GM; Figures 2c and d) but was sharply downregulated when cells underwent differentiation, as indicated by the appearance of myogenin transcripts (Figure 2b). In addition, the expression level of miR-203 in 100% GM was a little higher than that in 50% GM (Figures 2c and d). These results indicated that miR-203 is expressed in proliferating chicken primary myoblasts, but such expression is sharply downregulated during myoblast differentiation.

### miR-203 inhibits cell proliferation and induces cell cycle arrest in myoblasts

**Figure 3:**
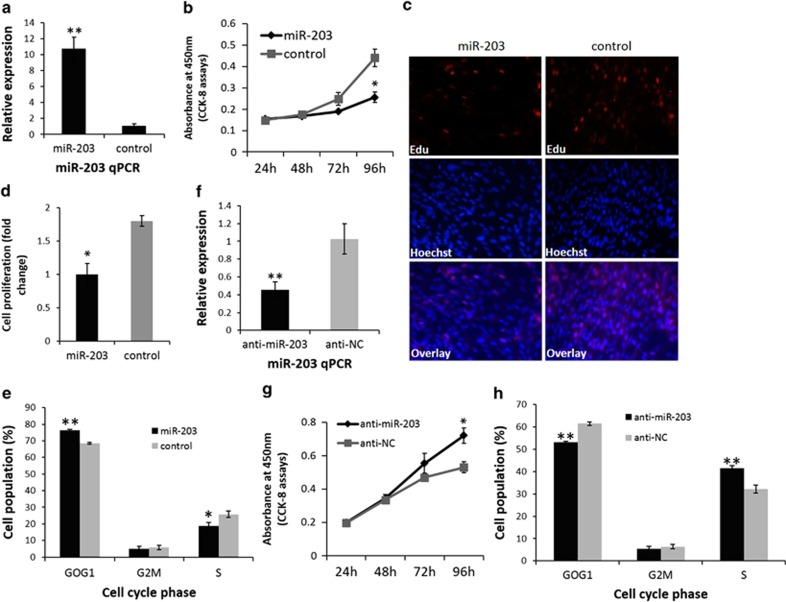
miR-203 represses chicken primary myoblast proliferation and induces cell cycle arrest. (**a**) miR-203 expression determined by qPCR in myoblasts transfected with miR-203 mimic or control duplexes. (**b**) Cell growth was measured following the transfection of miR-203 mimic or control duplexes into primary myoblasts in GM at 24, 48, 72 and 96 h. (**c**) The number of EdU-stained cells is significantly reduced after transfection with the miR-203 mimic. (**d**) The proliferation rate of myoblasts transfected with miR-203 is significantly reduced compared with that of control myoblasts. (**e**) Primary myoblasts were collected for cell cycle analysis 48 h after transfection. Propidium iodide staining for DNA content and a FACSAriaII flow cytometer were used to determine the percentage of cells in G0/G1, S and G2/M. (**f**) miR-203 expression determined by qPCR in myoblasts transfected with anti-miR-203 or anti-NC. (**g**) Cell growth was measured following the transfection of anti-miR-203 or anti-NC into primary myoblasts in GM at 24, 48, 72 and 96 h. (**h**) Primary myoblasts were collected for cell cycle analysis 48 h after transfection. Propidium iodide staining for DNA content and a FACSAriaII flow cytometer were used to determine the percentage of cells in G0/G1, S and G2/M. Results are expressed as the mean±S.E.M. of three replicates. (*P<0.05; **P<0.01)

To observe the effects of miR-203 on myoblast proliferation, we transfected myoblasts cultured in GM with an miR-203 mimic or scrambled negative control duplexes (Figure 3a), and then monitored the proliferation status of cells using the WST-8 assay and Cell-Counting Kit-8. miR-203 was shown to significantly inhibit myoblast proliferation (Figure 3b). 5-Ethynyl-2'-deoxyuridine (EdU) staining also demonstrated that the proliferation rate of miR-203-transfected cells was significantly reduced compared with that of the control cells (Figures 3c and d). Furthermore, flow cytometry analysis of the cell cycle revealed that myoblasts transfected with the miR-203 mimic could arrest myoblasts in the G0/G1 stage. The number of cells in the G0/G1 stage in the miR-203-transfected group was significantly higher than that in the control group (Figure 3e and Supplementary File 4). In addition, miR-203 loss-of-function by antagomir-miR-203 (anti-miR-203; Figure 3f) increased myoblast proliferation (Figure 3g) and reduced the number of cells in the G0/G1 stage (Figure 3h and Supplementary File 5). Together, the data suggest that miR-203 can represses myoblast proliferation and induces cell cycle arrest.

### miR-203 represses myoblast differentiation

**Figure 4:**
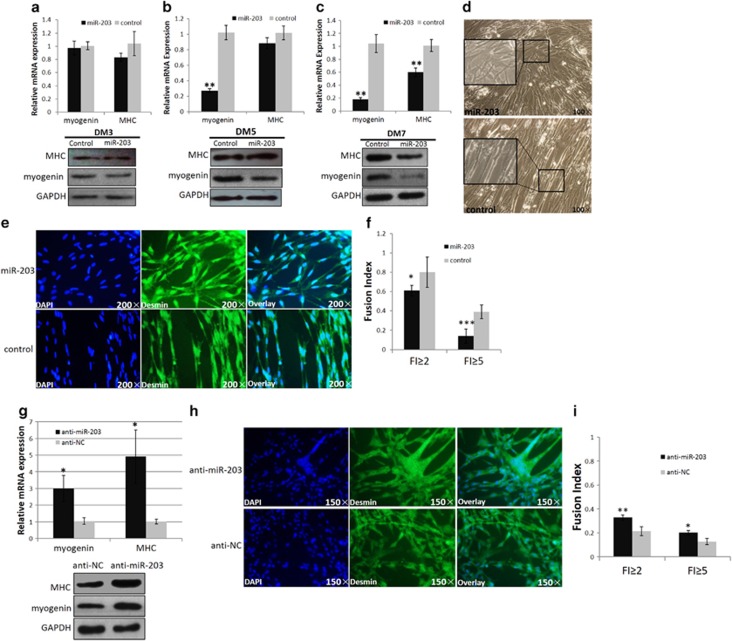
miR-203 represses myoblast differentiation. (**a**) After cells were transfected with miR-203 mimic at GM and collected at DM3, the mRNA and protein expression of *MHC* and *myogenin* do not change. (**b**) After cells were transfected with miR-203 mimic at DM2 and collected at DM5, the mRNA and protein expression of *myogenin* is significantly reduced, whereas the expression of *MHC* is slightly reduced. (**c**) After cells were transfected with miR-203 mimic at DM4 and collected at DM7, the mRNA and protein expression of *myogenin* and *MHC* are both significantly reduced. (**d**) Microscopic images of myoblasts at DM7 that were transfected at DM4. After miR-203 transfection, many myoblasts remain mononucleated and do not fuse to each other. (**e** and **f**) Cells were fixed and immunostained for desmin after 3 day of miR-203 and control duplex transfection at DM4, and then were quantified for the desmin^+^ nuclei presented in cells with the indicated number of nuclei (**f**). (**g**) After cells were transfected with anti-miR-203 at GM and collected at DM3, the mRNA and protein expression of *MHC* and *myogenin* are both significantly increased. (**h** and **i**) Cells were fixed and immunostained for desmin after 3 days of anti-miR-203 and anti-NC transfection at GM, and then were quantified for the desmin^+^ nuclei presented in cells with the indicated number of nuclei (**f**).The nuclei were stained blue with DAPI. The results are expressed as the mean±S.E.M. of three replicates. *P<0.05; **P<0.01; ***P<0.001

As cell cycle arrest is a critical step in the myoblast differentiation process and miR-203 is likely to be implicated in myoblast differentiation, we studied the effects of miR-203 on myoblast differentiation. Primary myoblasts cultured in GM were transfected with the miR-203 mimic or control duplexes. After transfection, the GM was replaced with differentiation medium to induce myoblast differentiation. Two major myoblast differentiation marker genes, *myogenin* and *MHC*, were detected 72 h after differentiation according to qPCR and western blot analyses. However, there were no differences in the expression levels of the cells transfected with the miR-203 and those transfected with the control duplex (Figure 4a). Considering that the myotubes formed before DM3 are fewer in number and small in size, the differentiation status and fusion index of these cells at this stage may be too low to identify the difference between these two transfected groups. Therefore, we subsequently performed the transfection at DM2 and observed the expression of *myogenin* and *MHC* at DM5. Under this condition, the expression of *myogenin* was significantly downregulated, and the expression of *MHC* was slightly lower in the miR-203-transfected myoblasts than that in the control myoblasts (Figure 4b).

Furthermore, we conducted transfection at DM4 and detected *myogenin* and *MHC* gene expression at DM7. *Myogenin* and *MHC* were both significantly downregulated in the miR-203-transfected myoblasts compared with the control myoblasts (Figure 4c). Myoblast fusion is a critical process during muscle differentiation. Therefore, we tested whether miR-203 transfection at DM4 could influence the fusion index of myoblasts at DM7. After miR-203 transfection, the myotubes were observed to be shorter and incomplete compared with those in the control myoblasts, and many myoblasts were not fused to nascent myotubes (Figure 4d). After immunofluorescence staining, we observed that the control myoblasts formed myotubes containing many nuclei, whereas the miR-203-transfected myoblasts remained mononucleated (Figure 4e). In addition, the fusion index of the miR-203-transfected myoblasts showed that miR-203 can significantly inhibit myoblast fusion (Figure 4f).

Furthermore, we also used anti-miR-203 and antagomir-NC (anti-NC) to test the impact of miR-203 loss-of-function during myoblast differentiation. The results showed that a significant increase in *myogenin* and *MHC* expression, as well as the fusion index, was observed, when miR-203 expression was inhibited (Figures 4g–i). Together, we argued that miR-203 can inhibit myoblast differentiation.

### *c-JUN* is a direct target of miR-203 in chickens

**Figure 5:**
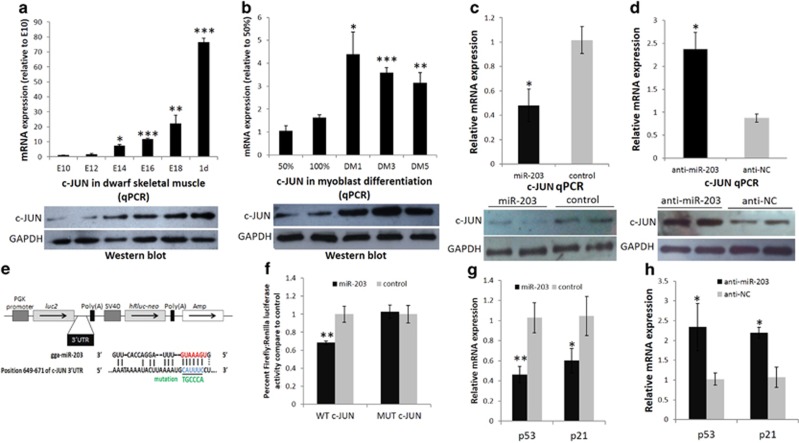
*c-JUN* is a direct target of miR-203 in chickens. (**a** and **b**) The mRNA and protein expression of *c-JUN* in the dwarf chicken embryonic leg muscle and primary myoblast differentiation process. (**c**) The mRNA and protein expression of *c-JUN* is significantly reduced after miR-203 transfection in myoblasts. (**d**) The mRNA and protein expression of *c-JUN* is significantly increased after anti-miR-203 transfection in myoblasts. (**e**) The *c-JUN* 3'-UTR was inserted into the dual-luciferase reporter vector pmirGLO (upper) at the 3' end of the firefly luciferase gene (*luc2*). Constitutive *Renilla* luciferase (*hRluc-neo*) expression was used as an internal normalisation control. The predicted binding site and mutated site (green) of miR-203 in the 3'-UTR of *c-JUN* is shown (bottom). (**f**) DF-1 cells were transfected with *c-JUN* 3'-UTR wild-type and mutant luciferase reporters and co-transfected with miR-203 mimic or control duplexes. The relative luciferase activity was measured 36 h later. (**g**) Relative *p53* and *p21* mRNA levels normalised to *β-actin* mRNA in myoblasts transfected with miR-203 mimic and control duplexes. (**h**) Relative *p53* and *p21* mRNA levels normalised to *β-actin* mRNA in myoblasts transfected with anti-miR-203 and anti-NC. All of the results are expressed as the mean±S.E.M. of three replicates. *P<0.05; **P<0.01; ***P<0.001

c-JUN is a positive regulator of cell proliferation,^20, 21^ and *c-JUN* is a direct target of miR-203 in basal cell carcinoma.^35^ Here, we studied the involvement of c-JUN in the inhibition of miR-203 during myoblast proliferation. *c-JUN* mRNA and protein level were upregulated from E10 to 1day in chicken skeletal muscle (Figure 5a), and they were also upregulated from the proliferation to the differentiation of myoblasts *in vitro* (Figure 5b). More importantly, the transfection of myoblasts with miR-203 in GM downregulated *c-JUN* mRNA and protein expression (Figure 5c), and the inhibition of endogenous miR-203 in GM using anti-miR-203 increased *c-JUN* mRNA and protein expression (Figure 5d).

To determine whether miR-203 can directly target *c-JUN*, we constructed two dual-luciferase reporters with the wild-type or mutant 3'-untranslated regions (3'-UTRs) of *c-JUN* inserted at the 3' end of the firefly luciferase gene (Figure 5e). Although the predicted miR-203 targeting sequence in the chicken 3'-UTR of *c-JUN* has one mismatch in the seed region (Figure 5e, bottom), the sequence also has two additional potential base pairings with miR-203 outside of the 7-mer seed region. When the dual-luciferase reporters were co-transfected with miR-203 mimic or control duplexes into DF-1 cells, miR-203 significantly reduced the firefly luciferase activity of the wild-type c-JUN reporter compared with the control duplexes (Figure 5f). Furthermore, when the predicted miR-203 seed region in the 3'-UTR was mutated, the mutant reporter no longer responded to miR-203 (Figure 5f). These results indicated that the predicted site is a target of miR-203 and is responsible for miR-203 targeting of the *c-JUN* 3'-UTR. Because the inhibition of cell proliferation by c-JUN is p53 dependent,^21^ we also detected the expression of *p53* and *p21* in miR-203 overexpression and loss-of-function myoblasts and control myoblasts. Both *p53* and *p21* expressions were significantly downregulated or upregulated with miR-203 overexpression or loss-of-function, respectively (Figures 5g and h). Therefore, we argued that *c-JUN* is a direct target of miR-203 in chickens.

### c-JUN promotes myoblast proliferation

**Figure 6:**
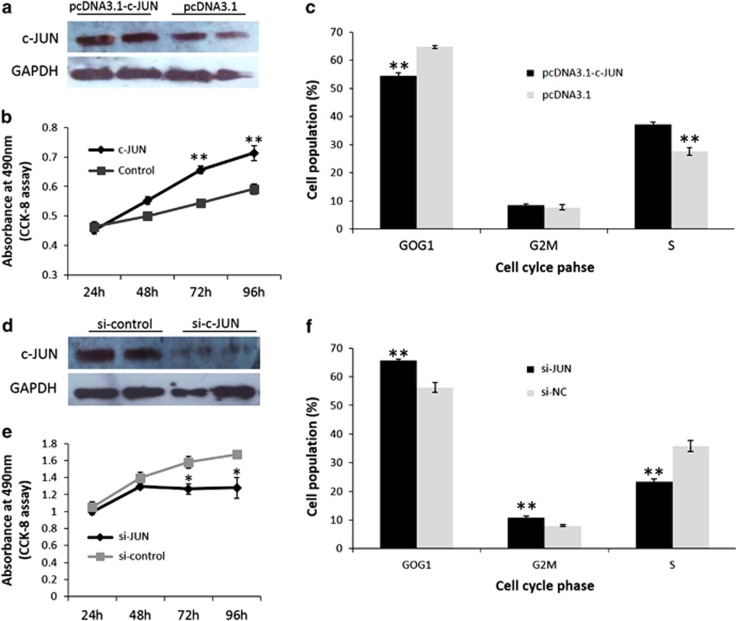
c-JUN promotes myoblast proliferation. (**a**) Overexpression of c-JUN via pcDNA3.1-c-JUN transfection increases the c-JUN protein level. (**b**) Cell growth is significantly increased after pcDNA3.1-c-JUN transfection compared with that in control cells. (**c**) Overexpression of *c-JUN* results in an increased cell population in the S phase and a decreased cell population in the G0/G1 phase. (**d**) Knockdown of *c-JUN* via si-c-JUN transfection reduces the c-JUN protein level. (**e**) Cell growth is significantly reduced after si-c-JUN transfection compared with that in control cells. (**f**) Knockdown of *c-JUN* results in an increased cell population in the G0/G1 and G2/M phases, and a decreased cell population in the S phase. All of the results are expressed as the mean±S.E.M. of three replicates. *P<0.05; **P<0.01; ***P<0.001

In this study, we performed *c-JUN* overexpression and knockdown experiments in primary myoblasts to verify the positive regulatory function of *c-JUN* in myogenic proliferation. As shown in Figures 6a and d, transfecting pcDNA3.1-c-JUN and si-c-JUN into myoblasts increased and reduced c-JUN protein, respectively. Overexpression of *c-JUN* significantly promoted myoblast proliferation (Figure 6b), reduced the number of cells that progressed to G0/G1 phase and increased the number of cells that progressed to S phase (Figure 6c and Supplementary File 6). Conversely, *c-JUN* knockdown significantly reduced myoblast proliferation (Figure 6e) and increased cell cycle arrest in the G0/G1 stage (Figure 6f and Supplementary File 7), a finding that is very similar to the effect of miR-203 overexpression. Together, these results showed that c-JUN can promote myoblast proliferation and this role is one reason for the inhibition of myoblast proliferation by miR-203.

### *MEF2C* is a direct target gene of miR-203 that has a positive role in myoblast differentiation

**Figure 7:**
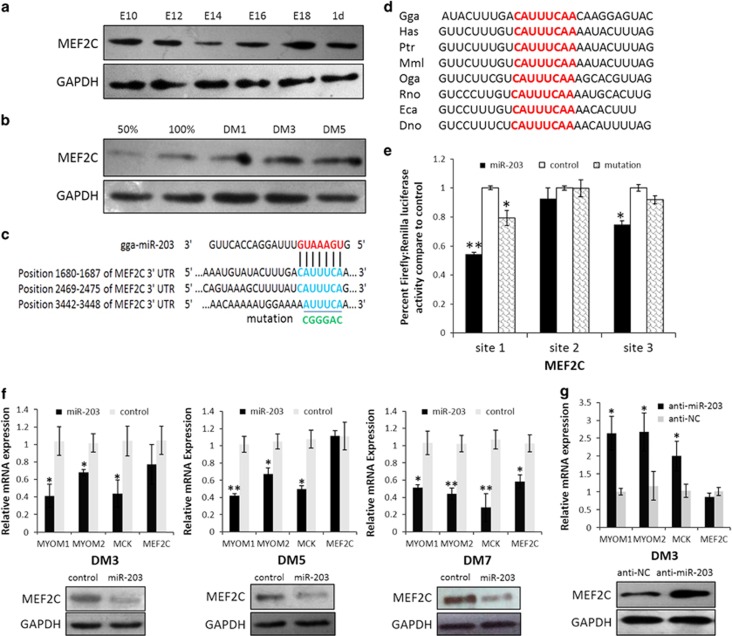

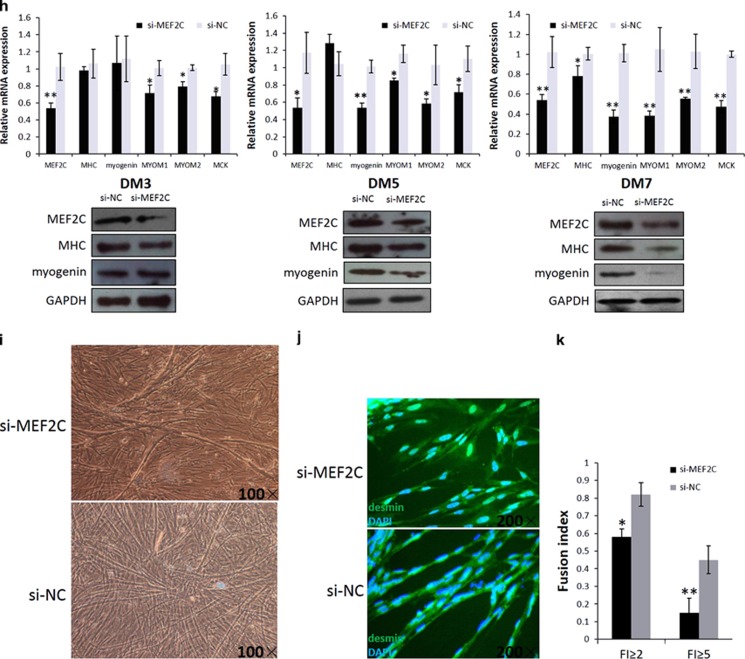
The negative role of miR-203 in skeletal muscle differentiation is carried out, in part, through the inhibition of *MEF2C* expression. (**a**) The expression of MEF2C protein during dwarf chicken leg muscle development at different embryonic days. (**b**) Expression of MEF2C protein during primary myoblast differentiation. (**c**) The predicted binding site and mutated site (green) of miR-203 in the 3'-UTR of *MEF2C*. (**d**) The predicted binding site 1 of miR-203 in the 3'-UTR of *MEF2C* is highly conserved among vertebrates. (**e**) Luciferase reporters were transfected into DF-1 cells with the miR-203 mimic or control duplexes, and luciferase activity was determined 36 h after transfection. (**f**) After 3 days of transfection, miR-203 significantly repressed MEF2C protein expression at DM3, DM5 and DM7. The mRNA levels of *MYOM1*, *MYOM2* and *MCK* are all decreased significantly. (**g**) After 3 days of transfection, anti-miR-203 significantly repressed MEF2C protein expression at DM3. The mRNA levels of *MYOM1*, *MYOM2* and *MCK* are all increased significantly. (**h**) Primary myoblasts were transfected with anti-MEF2C siRNA or negative control duplexes at GM, DM2 and DM4, respectively, and then were incubated in differentiation medium for 3 days before qPCR and immunoblotting. (**i**) Microscopic images of myotubes at DM7 that were transfected with si-MEF2C or negative control duplexes at DM4. (**j** and **k**) Cells were fixed and immunostained for desmin after 3 days of si-MEF2C and negative control duplex transfection at DM4, and then were quantified for the desmin^+^ nuclei presented in cells with the indicated number of nuclei. All of the results are expressed as the mean±S.E.M. of three replicates. *P<0.05; **P<0.01; ***P<0.001

*c-JUN* is a direct target of miR-203; however, no study has reported its relationship with myogenic differentiation. The overexpression of *c-JUN* in myoblasts had no impact on *myogenin* and *MHC* expression (Supplementary File 8). Therefore, we argued that there may be another target of miR-203 that functions in myogenic differentiation. Using TargetScan Release 6.2, we found that *MEF2C* is the most attractive candidate because it has well-established roles in skeletal myogenesis. The protein expression of *MEF2C* was downregulated during E14 and E16 (Figure 7a), and it was upregulated from proliferation to differentiation of myoblasts (Figure 7b), demonstrating that *MEF2C* expression correlated with miR-203 levels *in vitro* and *in vivo*. Furthermore, the *MEF2C* 3'-UTR has three predicted miR-203 binding sites (Figure 7c), and site 1 is highly conserved among vertebrates (Figure 7d). The dual-luciferase reporter assay verified that sites 1 and 3 could be bound by miR-203, whereas the seed site mutant reporters attenuated these interactions (Figure 7e). More importantly, miR-203 overexpression at different myoblast differentiation stages significantly inhibited the MEF2C protein level but not the *MEF2C* mRNA level (Figure 7f), and the expression levels of three muscle-specific genes, *MYOM1* (myomesin 1), *MYOM2* (myomesin 2) and *MCK* (muscle creatine kinase), which are directly regulated by MEF2C,^15^ were all significantly downregulated (Figure 7f). In addition, miR-203 loss-of-function increased MEF2C protein level but not mRNA level, and the three genes were all upregulated as compared with controls (Figure 7g).

We also used an anti-MEF2C siRNA to verify the myogenic roles of *MEF2C* in chicken primary myoblasts, and the siRNA and negative control duplexes were also transfected at three different stages. As shown in Figure 7h, si-MEF2C transfection significantly reduced the mRNA and protein levels of *MEF2C*, and the mRNA expression levels of three genes, *MYOM1*, *MYOM2* and *MCK,* were all significantly reduced. However, the expression of *myogenin* and *MHC* at DM3 was the same with si-MEF2C or control transfection, whereas *myogenin* expression was significantly reduced at DM5. The expression levels of both *myogenin* and *MHC* were both significantly reduced at DM7 (Figure 7h). In addition, reduced *MEF2C* expression also decreased the length and further formation of myotubes at DM7 (Figure 7j), along with a reduction of the fusion index (Figure 7k). Thus, *MEF2C* is a target of miR-203, and its knockdown significantly represses the late stage of myoblast differentiation.

## Discussion

**Figure 8:**
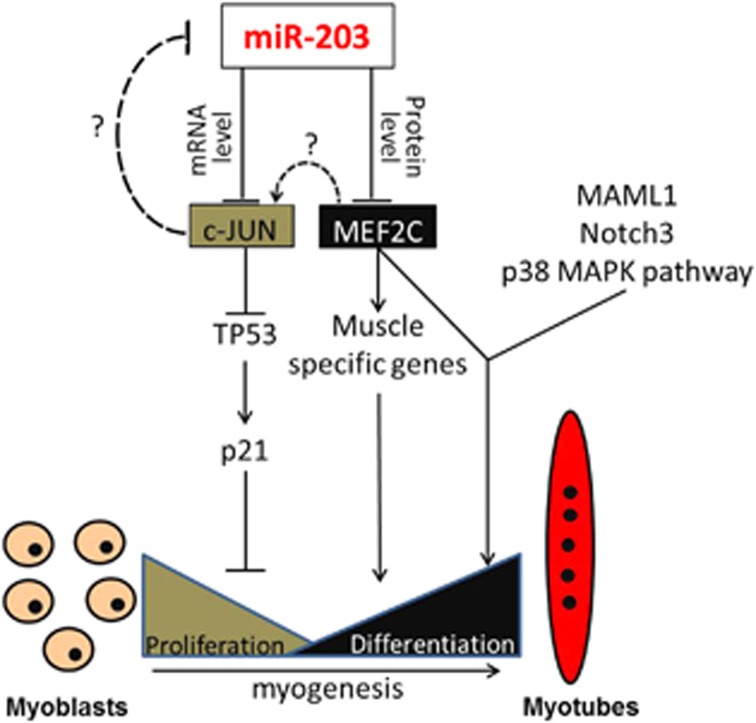
Model of the miR-203-mediated regulation network for skeletal muscle cell proliferation and differentiation. miR-203 inhibits the mRNA and protein level of *c-JUN* and *MEF2C*, respectively. The inhibitory role of c-JUN in cell proliferation is p53 dependent. MEF2C is involved in many muscle-specific gene transcription networks, and MEF2C can interact with MAML1, Notch3 and the p38 MAPK pathway to promote muscle differentiation. The dashed lines represent possible regulatory roles of MEF2C and c-JUN based on previous studies

In the present study, we demonstrated a transient expression pattern of miR-203 during embryonic skeletal muscle development and presented a role for miR-203 in myoblast proliferation and differentiation (Figure 8). miR-203 was previously described as a skin-abundant miRNA with an important role in skin differentiation,^28^ and the annotation of miR-203 in miRBase shows that it is a skin-specific miRNA.^36^ However, in the current study and our previous data,^33^ we found that miR-203 is abundantly expressed at E14 and E16 in chicken skeletal muscle. Another study also detected miR-203 expression in chicken embryo skeletal muscle.^32^ This skin-specific miRNA can also be expressed in early skeletal muscle cells, a finding that was similar to the three muscle-specific miRNAs miR-1, miR-133a and miR-206 that are expressed in brown pre- and mature adipocytes.^37^ Many miRNAs are not detected during early development but are expressed and functional during segmentation and differentiation.^38^ The most notable myomiRs, miR-206, miR-1 and miR-133a, are all significantly upregulated during muscle differentiation.^39, 40^ In our study, miR-203 is transiently upregulated at E10–E16 in chicken embryo leg muscle but is not detected after birth, indicating its potential role in muscle development.

The expression of myomiRs is often associated with phenotypic changes in skeletal muscle.^25, 41^ miR-203 was shown to exhibit higher expression in E14 dwarf chickens and in layers than in E14 normal chickens and in broilers.^32^ In addition, its upregulated expression in skeletal muscle correlated with slow muscle fibre formation and low muscle weight increase in the present study. The latter correlation was further validated using a series of *in vitro* miR-203 overexpression, loss-of-function and functional testing experiments. The expression of *c-JUN* and *MEF2C* correlated well with miR-203 expression during myoblast differentiation. In addition, subsequent miR-203 overexpression results showed that miR-203 can significantly inhibit myoblast proliferation and differentiation by repressing *c-JUN* and *MEF2C* expression.

miR-203 is widely recognised as an inhibitor of proliferation in various cancer lines by repressing various target genes.^27, 30, 42, 43, 44, 45, 46^ We found here that miR-203 can also inhibit myoblast proliferation by repressing *c-JUN* expression, an established target of miR-203 in human basal cell carcinoma.^35^ In addition, *p63*, another target of miR-203 in skin differentiation and in RMS cells,^28, 29, 30, 47^ was inhibited in miR-203-overexpressed myoblasts (Supplementary File 9). *p63* is important for cell proliferation in various tumours.^29, 47, 48^ Its knockdown represses myogenic differentiation,^49^ suggesting another possible regulation pathway of miR-203 in myoblast proliferation and differentiation. Collectively, miR-203 inhibits myoblast proliferation, in part, by repressing *c-JUN* expression.

In RMS cells, re-expression of miR-203 can induce myogenic differentiation by targeting *p63* and *LIFR*.^30^ However, *p63* was found to be a positive regulator of myogenic differentiation in another research, which was a study in C2C12 cell.^49^ According to our results, overexpression of miR-203 supressed myoblast differentiation, suggesting that the role of miR-203 may be different between RMS cells and myoblast. These findings were similar to the roles of miR-181s, which played a totally different role in different types of cancer cell.^50^ The downregulated expression of miR-203 from myoblast proliferation to differentiation is similar to that observed for miR-155, miR-125, miR-669a and miR-669q,^2, 51, 52^ which all act to repress skeletal muscle differentiation. *MEF2C*, a new target of miR-203 found in this study, is upregulated during C2C12 differentiation,^53^ and its interaction with MAML1, Notch3 and the p38 MAPK pathway is required for normal myogenesis (Figure 8).^16, 17, 18^ miR-135 and miR-133 can bind to the 3'-UTR of *MEF2C* and *MAML1*, respectively, repress the expression of MEF2C and MAML1, thus inhibiting myoblast differentiation.^53^ In our study, the MEF2C protein level was also upregulated during myoblast differentiation, and the overexpression of miR-203 repressed the MEF2C protein level and myoblast differentiation. Knockdown of *MEF2C* expression mimicked the overexpression of miR-203 by inhibiting the differentiation of myoblasts. In addition, one consequence of MEF2C activation is to increase the transcription of *c-JUN* in C2C12 cells and inflammatory cells,^54, 55^ and the overexpression of *c-JUN* suppresses miR-203 expression.^56^ Although our results do not verify the possibility in chicken myoblasts, they provide another source of evidence to support the regulation network of miR-203, *c-JUN* and *MEF2C* during myoblast differentiation into myotubes (Figure 8).

In conclusion, the embryonic expression pattern of miR-203 during skeletal muscle development in chickens is transient, and another important role of miR-203 is to repress muscle proliferation and differentiation. This repression, at least in part, is achieved by inhibiting *c-JUN* and *MEF2C* expression.

## Materials and Methods

### Animals

The hatching eggs of dwarf and normal recessive White Rock chickens, which were both bred for nearly 10 generations, were used in this study. More than four female embryos for each group were selected for leg muscle separation, and the sex of the embryos was determined by PCR amplification using sex-specific primers. With the skin and bones removed, the weight of one of the leg muscles was immediately measured using a precision balance (Sartorius, Gottingen, Germany; sensitivity 0.1 mg), and the leg muscle was then divided into three parts. The divided parts were frozen in liquid nitrogen and stored at −80 °C for use in DNA, RNA and protein extraction procedures. The other leg muscle sample of embryos was stored in 4% paraformaldehyde for histological analysis. Chickens were killed as necessary to ameliorate suffering.

### Histology

After fixation with 4% paraformaldehyde, the leg muscle samples were embedded in paraffin, and 10-*μ*m thick serial sections were made. The sections were then stained with H-E stain following standard protocols. Microscopic observation was performed with Carl Zeiss Primo Star microscope (Carl Zeiss, Oberkochen, Germany). Photographs were taken with a Moticam 2306 CCD imaging system (Motic Instruments lnc., CA, USA).

### Cell culture

#### DF-1 cell culture

DF-1 cells were cultured in high-glucose Dulbecco's modified Eagle's medium (Gibco, Grand Island, NY, USA) with 10% (v/v) foetal bovine serum (Hyclone, Logan, UT, USA) and 0.2% penicillin/streptomycin (Invitrogen, Carlsbad, CA, USA).

#### Chicken primary myoblast isolation and culture

Chicken primary myoblasts were isolated from the leg muscle of E10 chickens and minced in GM consisting of RPMI-1640 medium (Gibco), 20% foetal bovine serum, 10% chicken embryo extract, 1% nonessential amino acids and 0.2% penicillin/streptomycin. To release single cells, the suspension was shaken by repetitive vortexing and filtered to remove large debris; the cells were then collected by centrifugation at 350 *g* and resuspended in GM. Serial plating was performed to enrich myoblasts and eliminate fibroblasts. The differentiation of myoblasts was induced by replacing 20% foetal bovine serum with 2% horse serum (Hyclone).

### RNA isolation, RT-PCR and quantitative real-time PCR

Total RNA was extracted from tissues or cells with RNAiso reagent (Takara, Otsu, Japan) and treated with DNase I (Takara). The integrity and concentration of RNA were assessed by denatured gel electrophoresis and NanoDrop 2000c (Thermo, Waltham, MA, USA). cDNA synthesis for mRNA was carried out using PrimeScript RT reagent Kit (Perfect Real Time) (Takara) for RT-PCR and qPCR. qPCR was carried out in an Bio-rad CFX96 Real-Time Detection system (Bio-rad, Hercules, CA, USA) using KAPA SYBR FAST qPCR Kit (KAPA Biosystems, Wobrun, MA, USA), and quantification was done as described.^57^ For miRNA quantification, Bulge-loop miRNA qRT-PCR Primer Sets (one RT primer and a pair of qPCR primers for each set) specific for gga-miR-203 and U6 were designed by RiboBio (RiboBio, Guangzhou, China). miRNA bulge-loop was reverse transcribed with the First-Strand cDNA Synthesis Kit (Fermentas, Burlington, CA, USA) and quantified by qPCR using KAPA SYBR FAST qPCR Kit (KAPA Biosystems) according to the indicated manufacturer's instructions.

### Northern blotting and *in situ* hybridisation assays for miR-203

Northern blotting of miR-203 and U6 were performed with miRNA Northern Blot Assay Kit (Signosis, lnc., Sunnyvale, CA, USA) following the manufacturer's instructions. *In situ* hybridisation in E14 dwarf chicken leg muscle tissue was performed essentially as described by Obernosterer *et al.*^58^ with the following modifications. The 12-*μ*m thick cryosections were fixed in 4% PFA for 15 min at room temperature, treated for 15 min with Proteinase K, prehybridized and hybridised at 42 °C (miR-203 and scramble), and final colour development was performed with NBT/BCIP (Roche, Basel, Switzerland).

### Immunoblotting and immunofluorescence

Immunoblotting was performed using standard procedures and antibodies against c-JUN (Santa Cruz Biotechnology, Santa Cruz, CA, USA), MEF2C (Santa Cruz Biotechnology), MHC (Developmental Studies Hybridoma Bank, Iowa City, Iowa, USA), myogenin (Biorbyt, Cambridge, UK) and GAPDH (Bioworld, St Louis Park, MN, USA). For immunofluorescence, primary myoblasts treated in 24-well plates were fixed in 4% formaldehyde for 20 min and then washed three times for 5 min each in PBS. The cells were then permeabilised with 0.1% Triton X-100 for 15 min and were blocked with goat serum for 1 h. After blocking, the cells were incubated with anti-desmin (Bioss, Bejing, China) overnight at 4 °C. The FITC-conjugated anti-rabbit IgG was incubated for 2 h at room temperature. The cell nuclei were visualised using DAPI staining (Beyotime, Jiangsu, China). The fusion index was calculated according to the following formula: Fusion index=number of desmin^+^ myoblasts with more than two or five nuclei in a field/total desmin^+^ myoblasts number in a field.

### Cell proliferation assay

#### Flow cytometry analysis of the cell cycle

Primary myoblast cultures in GM were collected 48 h after transfection and then fixed in 75% ethanol overnight at −20 °C. After incubation in 50 *μ*g/ml propidium iodide (Sigma Life Science, St. Louis, MO, USA) containing 10 *μ*g/ml RNase A (Takara) and 0.2% (v/v) Triton X-100 (Sigma) for 30 min at 4 °C, the cells were analysed using a FACSAriaII flow cytometer (BD Biosciences, San Jose, CA, USA) and ModFit Lt 4.1 software (Verity Software House, Topsham, ME, USA).

#### EdU assays

Twelve hours after transfection, primary myoblasts were exposed to 10 *μ*M 5-ethynyl-2'-deoxyuridine (EdU; RiboBio) for 24 h at 37 °C. Next, the cells were fixed in 4% PFA for 30 min and permeabilised with 0.5% Triton X-100. Subsequently, 1 × Apollo reaction cocktail (RiboBio) was added to the cells and incubated for 30 min, and then the cells were stained with Hoechst 33342 for 30 min for DNA content analysis. Finally, the EdU-stained cells were visualised under a fluorescence microscope (Nikon, Tokyo, Japan). The analysis of myoblast proliferation (ratio of EdU^+^ to all myoblasts) was performed using images of randomly selected fields obtained on the fluorescence microscope. Assays were performed three times using triplicate wells.

#### CCK-8 assays

The transfected primary myoblasts were seeded at 1 × 10^3^ cells per well in a 96-well plate and cultured in GM for 4 days. Every 24 h, we added 10 *μ*l of Cell-Counting Kit-8 (CCK-8) reagents (Dojindo Laboratories, Kumamoto, Japan) to the cells for 2 h and then measured the absorbance at 450 nm using a Model 680 Microplate Reader (Bio-Rad).

### RNA oligonucleotides and transfection

The miR-203 mimics, mimic control duplexes, antagomirs of anti-miR-203 and anti-NC were purchased from RiboBio. siRNA against chicken c-JUN and MEF2C were from GenePharma (GenePharma, Suzhou, China), and a nonspecific duplex was used as the control. Transfection was performed with the Lipofectamine 2000 reagent (Invitrogen) combined with 50 nM of miRNA mimics, 200 nM of antagomir or 100 nM of siRNA and the procedure was performed according to the manufacturer's instructions.

### Plasmids construction

*pcDNA-3.1-c-JUN expression vector.* The *c-JUN* coding sequence was amplified from chicken leg muscle cDNA using PCR and the following specific primers: 5′-CCGCTCGAGGCCACCATGGAGCCTACTTTCTACGAG-3′ and 5′-CGGGGCCCCGCTTCTACCGTCAGCTTTAC-3′. The PCR product was cloned into the pcDNA-3.1 vector (Promega, Madison, WI, USA) using the *Xho*I and *Apa*I restriction sites.

#### pmirGLO dual-luciferase miRNA target expression vector

The 3′-UTRs of *c-JUN* and *MEF2C* were amplified from the chicken genome and cloned into the pmirGLO dual-luciferase reporter vector (Promega) using the *Dra*I and *Sal*I restriction sites. Because the 3′-UTR of *MEF2C* has three predicted miR-203 binding sites that are far away from each other, we generated three pmirGLO reporter vectors of the *MEF2C* 3′-UTR. The mutant c-JUN 3'-UTR and MEF2C 3′-UTR plasmids were generated by changing the miR-203 binding site from ATTTCA to CGCGGC, and mutagenesis was performed by PCR amplification and *Dpn*I digestion to remove the parental DNA.

### Dual-luciferase reporter assay

DF-1 cells were co-transfected with 100 ng of the wild-type or mutant 3'-UTR dual-luciferase reporter and 50 nM of the miR-203 mimic or negative control duplexes using Lipofectamine 2000 reagent in 96-well plates. After transfection for 48 h, the activities of firefly and *Renilla* luciferase were analysed using a dual-luciferase reporter assay system (Promega) following the manufacturer's instructions. The luminescent signal was quantified using a Fluorescence/Multi-Detection Microplate Reader (Synergy 2, Biotek, Winooski, VT, USA) and analysed with Gene5 software (Biotek, Bad Friedichshall, Germany).

### Statistical analysis

All results are presented as mean±S.E.M. based on at least three replicates for each treatment. Unpaired Student's *t*-test was used for *P*-value calculations.

### Ethics standards

The experiments in this study were approved by Animal Care Committee of South China Agricultural University (Guangzhou, China) with approval number SCAU#0014, and the chickens were humanely killed as necessary to ameliorate their suffering.

## Abbreviations

miRNAs: microRNAs
myomiRs: myogenic miRNAs
UTR: untranslated region
qPCR: quantitative polymerase chain reaction
EdU: 5-Ethynyl-2'-deoxyuridine
MEF2: myocyte enhancer factor 2
H-E: Haematoxylin-Eosin
MYOM1: myomesin 1
MYOM2: myomesin 2
MCK: muscle creatine kinase
