# Supplementary material for: The transient expression of miR-203 and its inhibiting effects on skeletal muscle cell proliferation and differentiation
Source: Cell Death Dis. 2014 Jul 17;5(7):e1347–. doi: 10.1038/cddis.2014.289 (PMC4123083; doi:10.1038/cddis.2014.289)
Supplement: Supplementary Information [file cddis2014289x1.pdf]

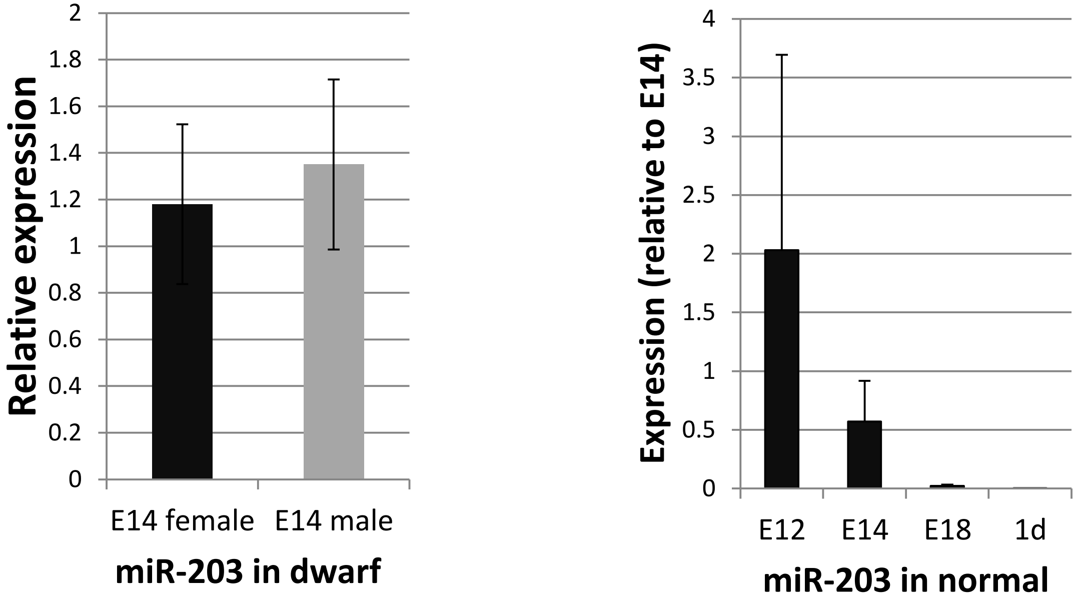

Supplementary file 1. miR-203 expression in normal chicken leg muscles, and in different sex of dwarf chickens leg muscles.

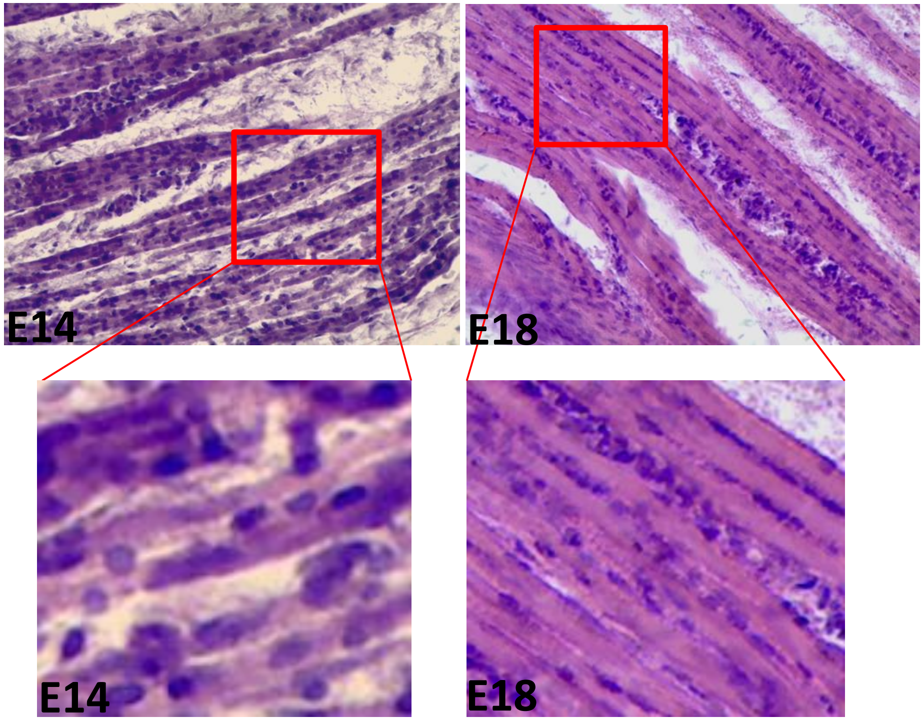

Supplementary file 2. H-E staining of leg muscle fiber vertical section of E14 and E18 dwarf chicken.

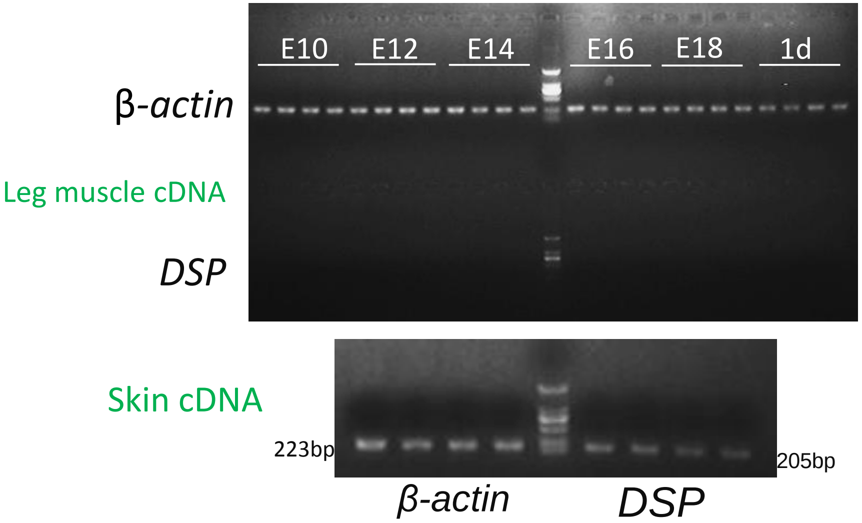

Supplementary file 3. RT-PCR validation that skin-specific gene *DSP* is not expressed in embryonic skeletal muscle samples. β-actin was used as the reference gene, skin cDNAs were used as reference samples.

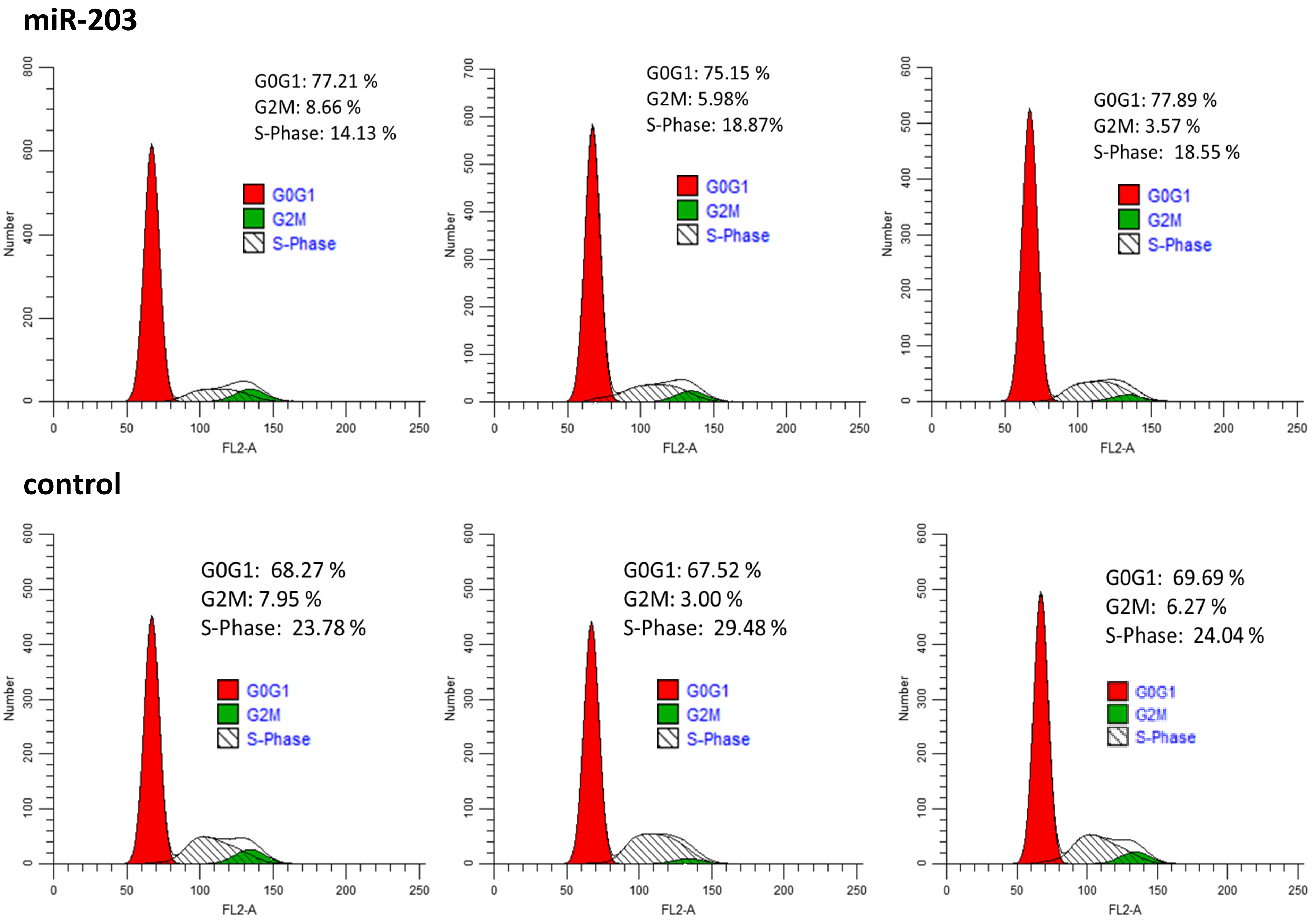

Supplementary file 4. The histograms of cell cycle analyzed by flow cytometry

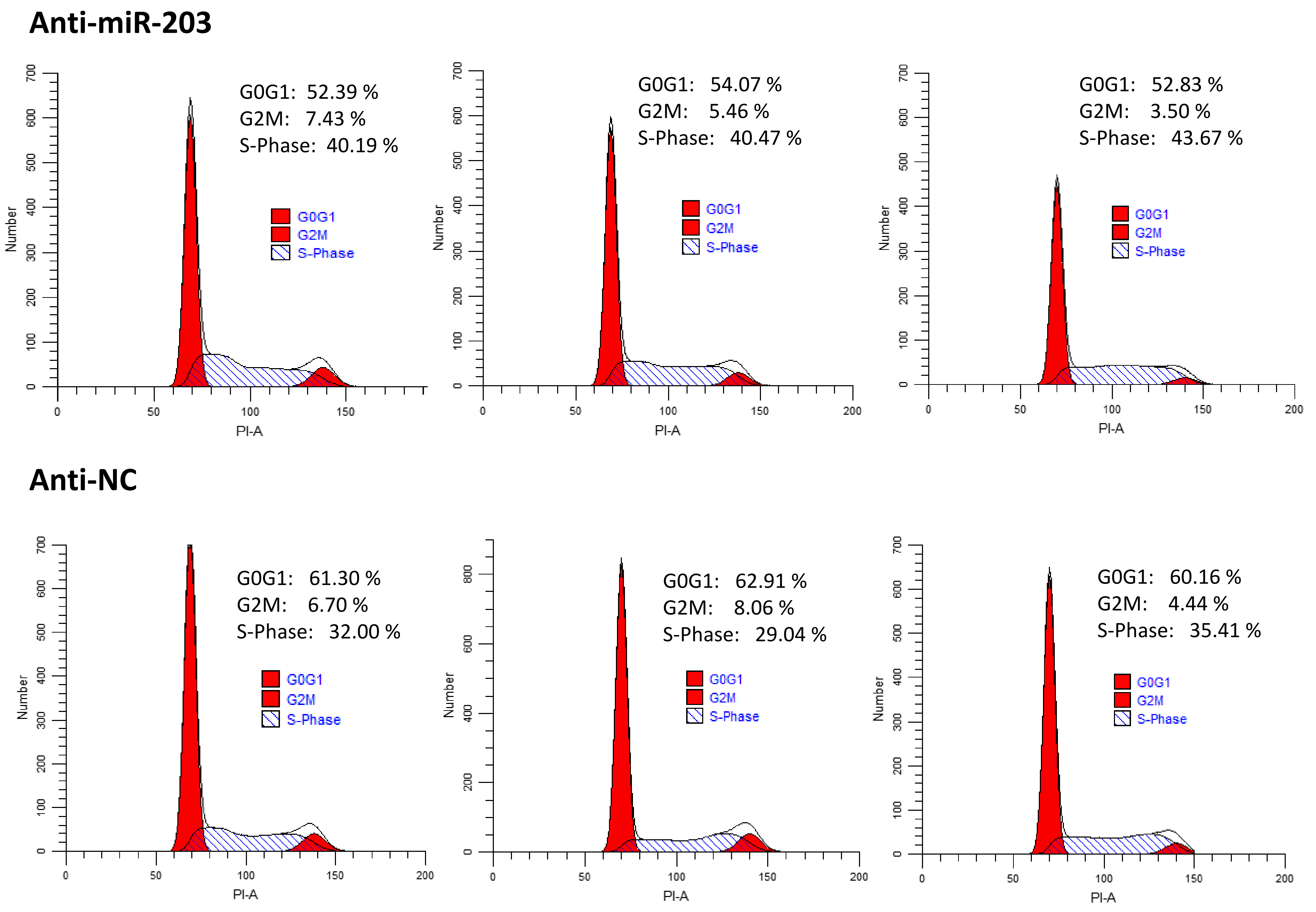

Supplementary file 5. The histograms of cell cycle analyzed by flow cytometry

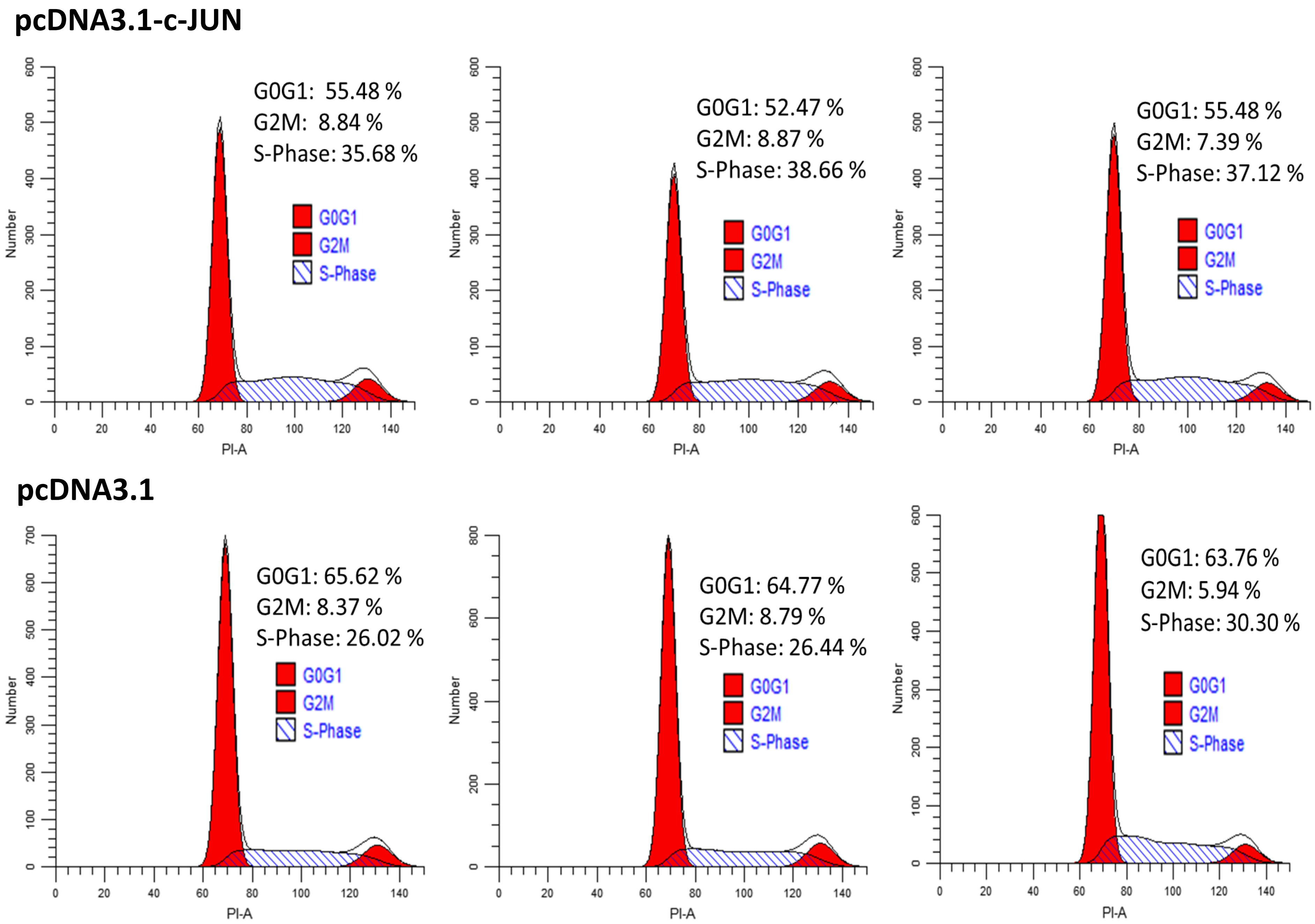

Supplementary file 6. The histograms of cell cycle analyzed by flow cytometry

si-c-JUN

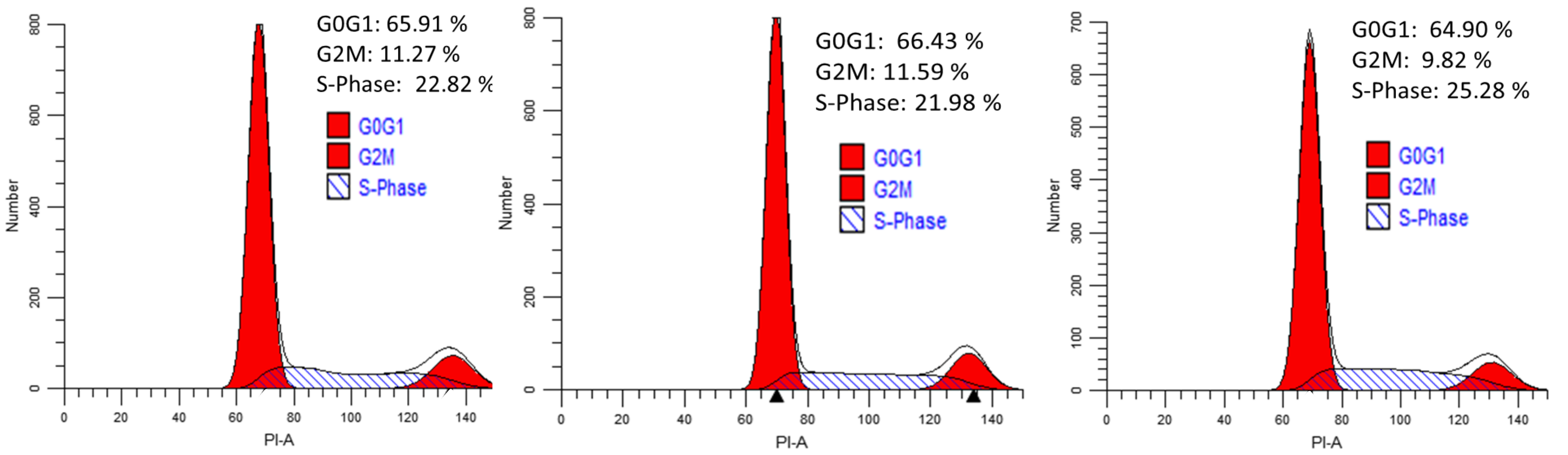

si-NC

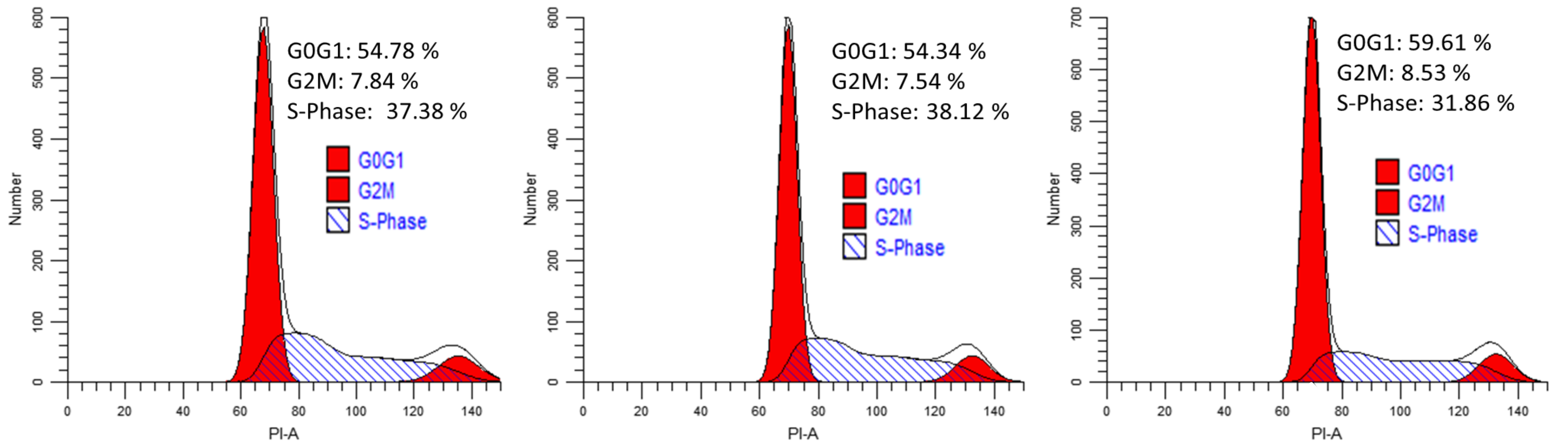

Supplementary file 7. The histograms of cell cycle analyzed by flow cytometry

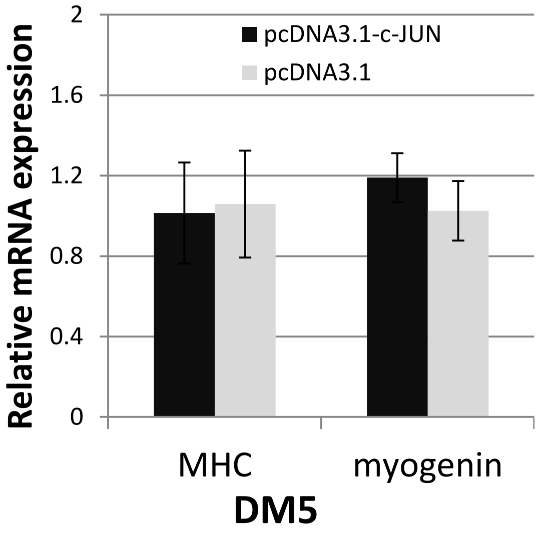

Supplementary file 8. Overexpression of *c-JUN* has no effect on the mRNA expression of *MHC* and *myogenin*.

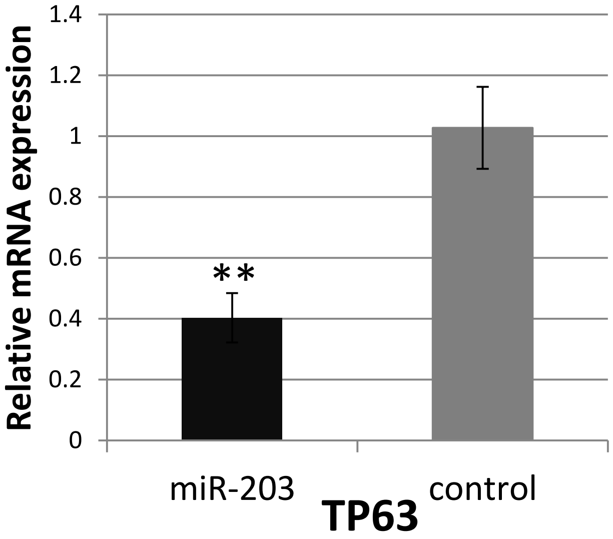

Supplementary file 9. miR-203 overexpression significantly inhibit *TP63* mRNA expression.
